# Flavonoids in natural products for the therapy of liver diseases: progress and future opportunities

**DOI:** 10.3389/fphar.2024.1485065

**Published:** 2024-10-24

**Authors:** Yanmei Liao, Fei Lv, Tianwen Quan, Chuan Wang, Jike Li

**Affiliations:** ^1^ Department of Pharmacy, Public Health Clinical Center of Chengdu, Chengdu, Sichuan, China; ^2^ Scientific Research and Teaching Department, Public Health Clinical Center of Chengdu, Chengdu, Sichuan, China

**Keywords:** liver disease, natural products, flavonoids, novel therapeutic drugs, hepatoprotective effect

## Abstract

The liver is the largest, important organ and the site for essential biochemical reactions in the human body. It has the function to detoxify toxic substances and synthesize useful biomolecules. Liver diseases related complications represent a significant source of morbidity and mortality worldwide, creating a substantial economic burden. Oxidative stress, excessive inflammation, and dysregulated energy metabolism significantly contributed to liver diseases. Therefore, discovery of novel therapeutic drugs for the treatment of liver diseases are urgently required. For centuries, flavonoids and their preparations which have the beneficial health effects in chronic diseases have been used to treat various human illnesses. Flavonoids mainly include flavones, isoflavones, flavanols, dihydroflavones, dihydroflavonols, anthocyanins and chalcones. The primary objective of this review is to assess the efficacy and safety of flavonoids, mainly from a clinical point of view and considering clinically relevant end-points. We summarized the recent progress in the research of hepatoprotective and molecular mechanisms of different flavonoids bioactive ingredients and also outlined the networks of underlying molecular signaling pathways. Further pharmacology and toxicology research will contribute to the development of natural products in flavonoids and their derivatives as medicines with alluring prospect in the clinical application.

## 1 Introduction

Liver disease and its complications are important sources of morbidity and mortality in the world, which have become a major economic burden and public health issue that cannot be ignored. Liver diseases resulting from acute or chronic etiologies, manifest different pathological steps from acute injury, toxicity and necrosis to fibrosis, cirrhosis, and cancer, which is a four-stage process that includes liver inflammation, liver fibrosis, cirrhosis, and liver failure. There are many causes of liver inflammation, the most common being viral factors such as hepatitis B and C. It can also be caused by bad habits such as fatty liver, which includes alcoholic and nonalcoholic fatty livers (Neshat et al., 2021). However, the treatment of these liver diseases is still challenging due to the difficulty of diagnosis in the early stage of the onset, insufficient understanding of the pathogenesis and limited treatment options. There are currently more than 1.3 billion liver disease patients worldwide, and viral hepatitis, especially hepatitis B, is still the main cause of chronic liver disease and end-stage liver disease. Once a patient has progressed to irreversible end-stage liver diseases, chemotherapy and liver transplantation can only be the only treatment options. Despite prominent advancements in modern medicine, there are still no dependable medications that may increase liver function, provide complete liver protection, or multiply liver cells (Madrigal-Santillán et al., 2014). The traditional Chinese medicines are widely used to treat liver diseases in China and other East Asian countries. When western medicines have no cure for chronic liver diseases, traditional Chinese medicines still play an important role. Accordingly, considerable research efforts have been devoted to identifying medicinal plants that have the ability to both prevent and treat liver diseases. More and more evidences show that several traditional Chinese medicine prescriptions have gratifying therapeutic effects on many chronic liver diseases (Barzegari et al., 2024; Chou et al., 2024). Thus, the active ingredients from these hepatoprotective herbal medicines may become a major source of novel drug discovery for the treatment of liver diseases.

**FIGURE 1:**
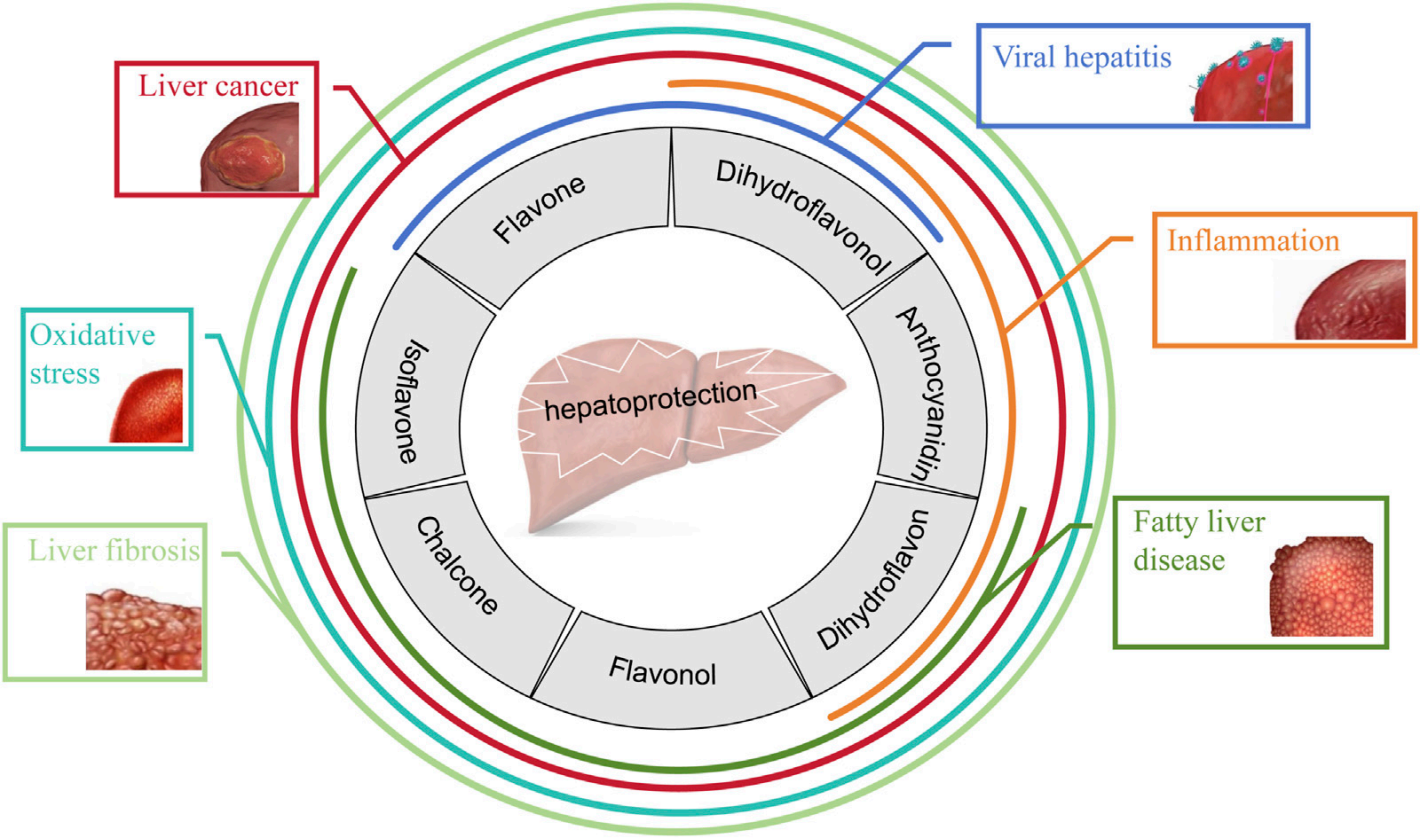
Hepatoprotective effects of seven classes of flavonoids.

In the past few decades, available data have suggested the therapeutic effects of various natural products on liver diseases, among which the performance of flavonoids is outstanding. Flavonoids are the secondary metabolites of many fruits, herbs, roots, stems, bark, flowers, grains, tea and wine, which not only impart color and protection to plants, but also make them safe to eat, so they are also called “Phytonutrients”. Flavonoids are compounds with the structure of 2-phenylflavonoids which are widely distributed in nature and are characterized by a variety of biological activities, including cardiovascular activity, antibacterial, antiviral, antitumor, antioxidant, antiinflammatory, and liver protection (Yang F. et al., 2024; Wang Q. et al., 2024; Yang et al., 2019). Figure 1 shows the common therapeutic effects of flavonoids in liver disease.

**FIGURE 2:**
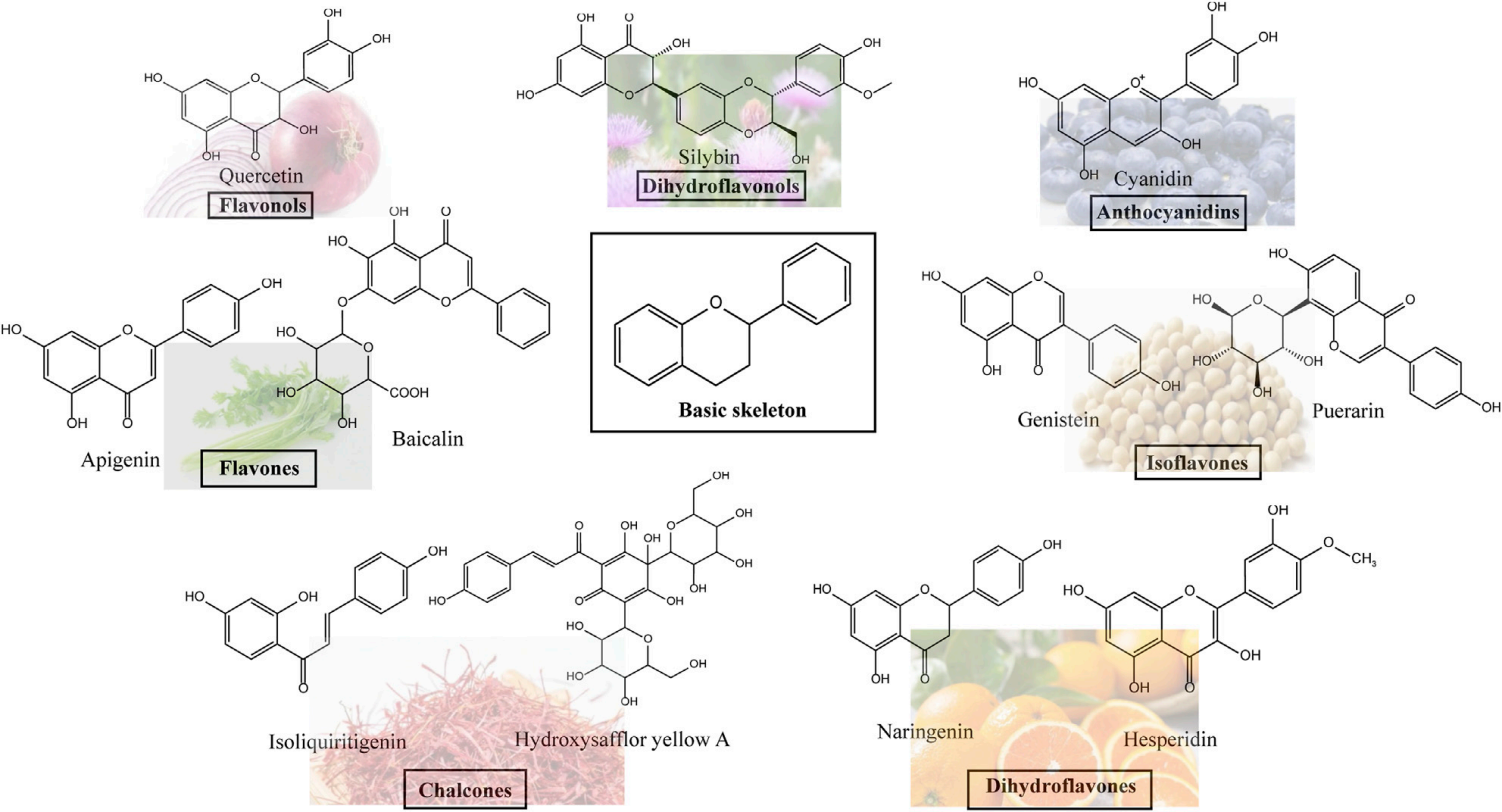
Chemical structures of Flavonoids compounds against liver disease.

**FIGURE 3:**
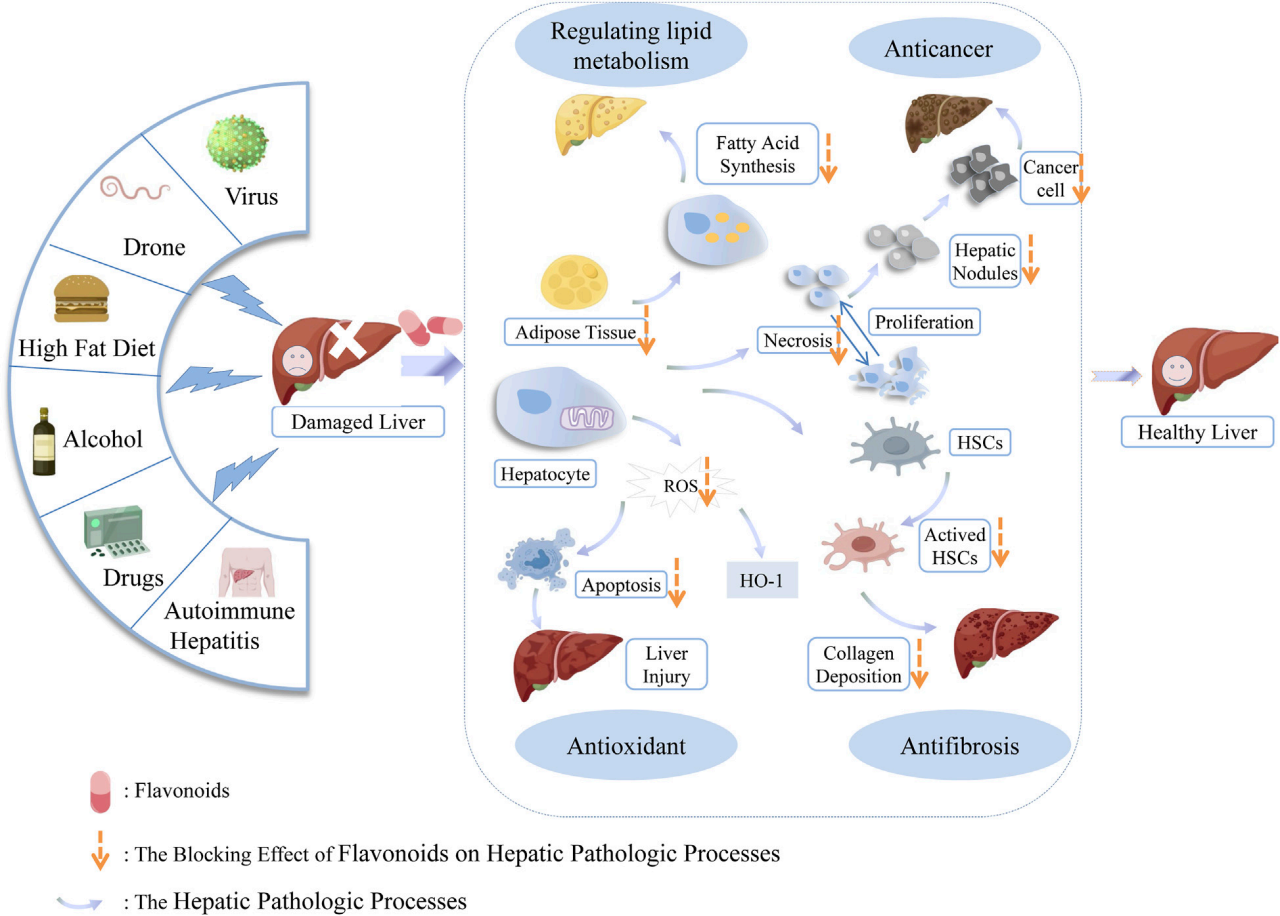
Developmental course of liver diseases and therapeutic role of flavonoids.

Flavonoids, which have a benzo-γ-pyrone structure with at least one hydroxyl group are polyphenolic compounds that are ubiquitously found in nature, with more than 4,000 identified varieties. Flavonoids are categorized into subclasses based on variations in the C ring. The major subclasses are flavones, anthocyanidins, isoflavones, chalcones, dihydroflavones, dihydroflavonols and flavanols (as described in detail in Table 1) (Ross and Kasum, 2002). Flavonoids comprise a variety of biologically active compounds, among which there were some components of various medicinal preparations to treat a few human illnesses over thousands of years. Figure 2 shows the chemical structures of major flavonoids with hepatoprotective effects. Evidence has shown the potential use of flavonoids in several ways such as against inflammation, oxidative stress, immunoregulation, virus infection, and cancer. During recent years, numerous researches have reported the hepatoprotective effects of flavonoids (Okada et al., 2017; Xu et al., 2019; Liu et al., 2021; Zhang et al., 2013), which is undoubtedly a gratifying discovery for the field of liver disease treatment. Oxidative stress, inflammatory disorders and energy metabolism dynamic balance disorders have been widely documented as potential mechanisms that mainly involve chronic and acute liver diseases. By complexing with oxidizing species, hydroxyl groups in flavonoids render these compounds the ability to scavenge and stabilize free radicals, reducing oxidative damage (Kumar and Pandey, 2013). Flavonoids have been used clinically in Japan and Europe, mainly for the treatment of chronic viral hepatitis (Kao et al., 2014; Yang et al., 2015; Pastorino et al., 2018; Michielsen et al., 2018). In this review, we summarize the effective role of flavonoids in the treatment of liver disease (in Figure 3), and provide insights into their mechanism of action and targets. In addition, we highlight the prospect of this biologically active ingredients as therapeutic drugs for clinical treatment of liver disease.

**TABLE 1:** The major subclasses of flavonoids.

Class of flavonoids	Chemical structure	Dietary source	Compound	Pharmacological effect	Reference
Flavones	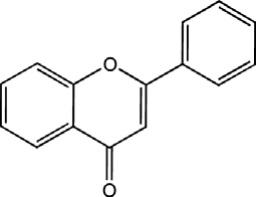	Celery, parsley	Baicalin, Baicalein, Apigenin	Anticancer, antioxidant, antihepatic fibrosis, antivirus	Fang et al. (2017), Zhou et al. (2017a)
Anthocyanidins	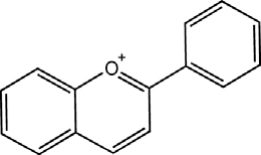	Cherry, strawberry, grapes, apples	Cyanidin, Peonidin, Petunidin	Anticancer, antioxidant, antihepatic fibrosis, antiinflammatory	Galvano et al. (2004), Mattioli et al. (2020)
Isoflavones	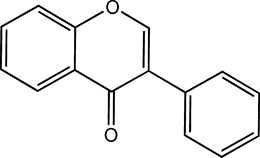	Soybeans and other legumes	Genistein, Puerarin	Amelioration of hepatic steatosis, anticancer, antioxidant, antihepatic fibrosis	Lee et al. (2009), Cao et al. (2017)
Chalcones	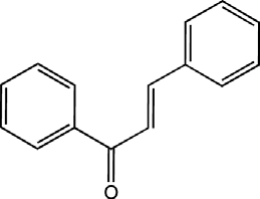	Fruits, grains, legumes, vegetables	Isoliquiritigenin, Hydroxysafflor yellow A	Amelioration of hepatic steatosis, anticancer, antioxidant, antihepatic fibrosis	Cao et al. (2016), Wang et al. (2021b)
Dihydroflavones	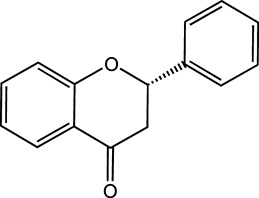	Oranges, lemons, grapes	Hesperidin, Naringenin	Amelioration of hepatic steatosis, anticancer, antioxidant, antihepatic fibrosis, antiinflammatory	Mesallam and Atef (2020), Shrikanth and Nandini (2020)
Dihydroflavonols	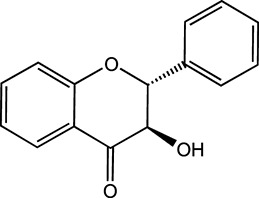	Milk thistle	Silybin	Anticancer, antioxidant, antihepatic fibrosis, antiinflammatory, antivirus	Loguercio and Festi (2011), Loguercio et al. (2012)
Flavonols	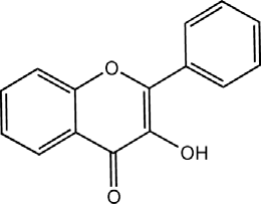	Onions, kale, lettuce, tomatoes	Quercetin	Amelioration of hepatic steatosis, anticancer, antioxidant, antihepatic fibrosis	Huang et al. (2013), Rani (2017)

## 2 Flavonoids in liver disease

Flavonoids have been widely studied for the treatment in liver disease. One of the most intensively studied flavonoids is silybin (SL), which is extracted from the milk thistle. In addition, the flavonoids in licorice have shown outstanding performance in the field of clinical treatment of liver diseases (Kim et al., 2015). Apigenin (AP) and Puerarin (PA) are also extensively studied (Feng et al., 2016; He et al., 2021). Together with quercetin (QE), which is contributed to the treatment of nonalcoholic fatty liver disease (NAFLD). The major subclasses of flavonoids including flavones, anthocyanidins, isoflavones, chalcones, dihydroflavones, dihydroflavonols and flavanols and their effects in liver disease are reviewed separately. In this article, the effects of these compounds will be considered, which in recent years, have been the target of research of worldwide interest.

### 2.1 Flavones

Flavones are one of the important subclasses of flavonoids, being widely found as glucosides in leaves, flowers and fruits, such as celery, parsley, red pepper, chamomile, mint and ginkgo. Baicalin and AP belong to this group of flavonoids.

#### 2.1.1 The flavonoids of baicalensis (BF)

A total of 56 free flavonoids have been isolated from baicalensis. The most abundant flavonoids are wogonin (5,7-dihydroxy-8-methoxyflavone), wogonoside (Wogonin-7-glucuronic acid), baicalin (7-glucuronic acid, 5,6-dihydroxy-flavone) and baicalein, of which the therapeutic values mainly lie in its therapeutic effects in various liver diseases including hepatitis, cirrhosis and liver cancer (Zhao et al., 2006).

##### 2.1.1.1 Effects of BF on hepatic oxidative stress

Zhang et al. investigated the effects of baicalin on iron overload-induced liver injury *in vivo* and found that it significantly inhibited iron overload-induced lipid peroxidation and protein oxidation of liver, and reduced hepatic iron and collagen content. This inhibitory effect on protein oxidation may come from a combination of iron eliminating and direct scavenging of free radicals. The nuclear factor erythroid 2-related factor 2 (Nrf2)/antioxidant response element (ARE) signaling pathway is an important mediator of cellular response during an oxidative stress condition (Zhang S. et al., 2006). Baicalin protected the liver against oxidative stress in hepatic mitochondria caused by duck hepatitis A virus type 1 (DHAV-1) via activating the Nrf2/ARE signaling pathway (Su et al., 2021). Wang et al. also showed the administration of the antioxidative effect of curcumin combined with baicalin on ethanol-induced oxidative damage in rat liver, which was closely related to Nrf2/haem oxygenase-1 (HO-1) pathway activation, and can improve ethanol-induced hepatitis by inhibiting p38 mitogen-activated protein kinase (MAPK) and tuberous sclerosis complex 1/eukaryotic initiation factor 2α/activating transcription factor 4 pathway (Wang Q. et al., 2020; Wang L. et al., 2021).

##### 2.1.1.2 Effects of BF on viral hepatitis

It has been shown that baicalin can inhibit hepatitis B virus (HBV) RNAs production and reduces levels of the related hepatocyte nuclear factors (HNFs). Xia et al. proposed a mechanism for anti-HBV activity of baicalin in an HNF4α-HNF1α-dependent manner, which diminished HNF4α and HNF1α transactivation, and effectively inhibited HBV transcription and virus replication (Xia et al., 2020). In the study by An et al. both OAG, a flavonoids produced by microbial conversion, and its substrate baicalin exhibited anti-HBV activity, inhibited secretion of HBsAg and HBeAg from HBV-infected HepG2 2.2.15 cells, and affected the release of viral DNA (An et al., 2017). Huang et al. explored the potential effects and mechanism of baicalin and entecavir (ETV), which is a first-line anti-HBV drug, in HBV-DNA, HBsAg/HBeAg seroconversion and drug-resistance. Compared with ETV monotherapy, co-treatment of baicalin with ETV could more effectively control HBV-DNA and viral protein secretion in hepG2.2.15 cell. And baicalin could significantly enhance the ETV’s efficacy in the nucleoside analogs-resistant HBVrtM204V/rtLl80M cell lines, indicating that baicalin had an advantage in overcoming the HBV virus drug-resistance mutation (Huang et al., 2017).

##### 2.1.1.3 Effects of BF on liver fibrosis

Clinically, liver fibrosis will gradually develop into liver cirrhosis, and it is likely to cause liver dysfunction and liver failure. Liver fibrosis is recognized as a complicated pathogenic mechanism with extracellular matrix accumulation and HSC activation. Natural products are potential resources for the development of agents to treat liver fibrosis. Baicalin has been reported to improve liver injury and fibrosis by suppressing transforming growth factor beta1 (TGFβ1), a major fibrogenic inflammatory cytokine, and activating peroxisome proliferator-activated receptor γ (PPARγ), a nuclear receptor with protective effect in fibrosis (Qiao et al., 2011). The therapeutic effect of baicalin on hepatic fibrosis may involve microRNA-122 (miR-122) and its target gene Kruppel-like transcription factor 6 (KLF6). It is demonstrated that baicalin dose-dependently restored the expression if miR-122 in the serum and liver of CCl4-treated mice. In addition, baicalin also reduced KLF6, a fibrogenic nuclear protein that is directly targeted by miR122 (Zhang et al., 2016). It has been shown that baicalensis strongly prevented hepatic fibrosis by activating ERK-p53 pathways, which in turn induced G2/M cell cycle arrest and activated caspases in hepatic stellate cells (HSCs) (Pan et al., 2015). Li et al. found that alanine aminotransferase (ALT) normalization rate, HBV-DNA and HBeAg negative conversion rate and anti-HBe seroconversion rate of the treatment group was treated with baicalin and telbivudine were significantly higher than the control group were treated with only telbivudine, and that post-treatment markers of hepatic fibrosis, hyaluronic acid, laminin, and type III procollagen, in the treatment group differed significantly from those in the control group (Li et al., 2011).

##### 2.1.1.4 Effects of BF on liver cancer

Baicalin exhibits high antitumor activity towards HBV-infected liver cancer. Fang et al. designed and synthesized multifunctional selenium nanoparticles (SeNPs) with baicalin and folic acid (FA) surface modifications for targeted therapy of HBV-infected HCC. Interestingly, baicalin-SeNPs-FA induced HepG2215 cell apoptosis by down-regulating the generation of reactive oxygen species (ROS) and the expression of the HBV X antigen (HBxAg) protein and exhibited an excellent ability to inhibit cancer cell migration and invasion (Fang et al., 2017). This is supported by Yu et al.‘s work demonstrating that baicalin induced apoptotic cell death in HepG2 and SMMC-7721 cells (HCC lines), which was accompanied by upregulation of Bax, downregulation of B-cell lymphoma-2 (Bcl-2), and cleavages of Caspase-9, Caspase-3, and poly (ADP-ribose) polymerase (PARP) (Yu et al., 2015). The anticancer properties of baicalein, including inhibition of adhesion, invasion, migration and proliferation of HCC cells. They observed that baicalein reduced the gelatinolytic activity of the matrix metalloproteinases (MMP)-2, MMP-9, and urokinase-type plasminogen activator (uPA), decreased p50 and p65 nuclear translocation, and also decreased the phosphorylation levels of protein kinase C (PKC)-α and p38 proteins. Furthermore, the suppression of invasion and metastasis in HCC by baicalein is strictly dependent on the hydroxyl substitution of its A-ring (C7) (Chiu et al., 2011). The cytotoxic effects of Scutellaria baicalensis and its flavonoids in HCC were comprehensively summarized by Chen et al., including 1) depletion of glutathione in HCC cells; 2) alteration of mitochondrial membrane potential and induction of cytochrome c release; 3) inhibition of mitogen-activated protein/extracellular signal-regulated kinase (MEK)-extracellular-signal-regulated kinase (ERK) signalling pathway and induction of intrinsic apoptosis; 4) attenuation of nuclear factor kappa B (NF-κB) activity and sensitizing tumor necrosis factor α (TNF-α) induced apoptosis by transferring electron free radicals (Chen et al., 2021).

##### 2.1.1.5 Clinical application of BF

Baicalin has long been studied clinically in combination with other drugs in liver disease. Another study in Sichuan Mianyang 404 hospital indicated that baicalin capsules combined with thymalfasin in treatment of chronic hepatitis B can obviously improve the symptoms, relieve liver injury, maintain immune balance, and has obvious anti-HBV effect (Liu and Liu, 2018). Baicalin capsules combined with adefovir tablets in the treatment of chronic hepatitis B resulted in a significantly superior efficacy index such as ALT normalized ratio, HBeAg-negative conversion rate, HBeAg/HBeAb seroconversion rate, and HBV-DNA-negative conversion rate compared with treatment with adefovir tablets only (Chen et al., 2010). In addition, there was also a study indicated that the combination of baicalin and interferon α-2b could improve the sera negative transformed rate and protect liver cells from inflammation and prohibitive liver fibrosis (Song et al., 2013).

#### 2.1.2 AP

AP, 4,5,7-trihydroxyflavone, is a flavonoid mainly derived from parsley and chamomile that has been used as an antioxidant and antiinflammatory agent for many years, and recorded as a traditional Chinese medicine to interfere with the various disease progression such as cancer (Madunic et al., 2018; Yan et al., 2017), inflammation (Kim and Lee, 2018), oxidation (Sanchez-Marzo et al., 2019). Especially, the anticancer properties of AP have been reported in variety of previous studies.

##### 2.1.2.1 Effects of AP on hepatic oxidative stress

The oxidative stress and inflammation have long been proposed as an important biochemical mechanism underlying the development of liver injury (Jaeschke, 2000). Therefore, the antioxidants may be beneficial for the prevention and cure of liver injury. Emerging evidence demonstrated that AP had potential antioxidative stress activity *in vitro* and *in vivo*. It has been reported that AP can alleviate the degree of liver injury induced by D-galactosamine (D-GalN)/lipopolysaccharide (LPS) by increasing the levels of hepatic Nrf-2 and PPARγ protein expressions as well as superoxide dismutase, catalase, glutathione s-transferase and glutathione reductase activities and decreasing the levels of hepatic NF-κB protein expression and TNF-α (Zhou R. J. et al., 2017). AP-7-glucoside isolated from I. chinesis had a protective effect against liver injury caused by CCl4 by decreasing the contents of malondialdehyde (MDA), glutamic pyruvic transaminase (GPT), glutamic oxaloacetic transaminase (GOT) and 8-Hydroxydeoxyguanosine, as well as increasing the contents of glutathione (Zheng et al., 2005). AP was first found to alleviate palmitic acid (PA)-induced NOD-, LRR-, and pyrin domain-containing 3 (NLRP3) inflammasome activation and pyroptosis in HepG2 cells and primary mouse hepatic cells, indicating that AP could alleviate PA-induced pyroptosis by activating autophagy (Meng et al., 2022).

##### 2.1.2.2 Effects of AP on liver cancer

This was demonstrated in a number of studies that AP exhibited anticancer activity in numerous HCC models. For the treatment of liver cancer, AP may suppress tumor growth of HCC through the long non-coding RNA H19-mediated Wnt/β-catenin signaling regulatory axis, which indicated that the H19 was a potential therapeutic target for HCC (Pan et al., 2021). Additionally, AP was found to inhibit self-renewal capacity significantly, and reduced the protein expression level of casein kinase 2α of liver cancer stem-like cells in a concentration-dependent manner (Jiang et al., 2017). Administration of AP (10, 20, and 40 μM) decreased the spheroid-forming ratio, upregulated expression of src homology region 2 domain-containing phosphatase 1 and suppressed the phosphorylation of signal transducers and activators of transcription 3 (STAT3) protein in human liver cancer MHCC97H cells in a dose-dependent manner (Cui et al., 2018). According to a recent study, combined treatment of sorafenib and AP could make more enhanced effects on HepG2 cell than single treatment groups. And the effects on migration, invasion, apoptosis and gene expressions showed that sorafenib and AP may have synergistic effect (Şirin et al., 2020). The potential of AP to enhance cisplatin’s chemotherapeutic efficacy was found in HepG2, Hep3B, and Huh7 liver cancer cell lines, suggesting that the combined treatment exhibited a significant antiinvasive and antimigratory action in all cancer cell lines (Papachristou et al., 2021). Ganguly et al. developed galactose-tailored poly (lactide-co-glycolide) (PLGA) nanoparticles loaded with AP (AP-GAL-NPs) for active liver targeting to treat HCC and found that AP-GAL-NPs exhibited a better protective effect against HCC in rats evidenced by the significant reduction of nodule formation, downregulation of MMP-2 and MMP-9, and induction of apoptosis in the liver (Ganguly et al., 2021). It has been shown that the significant anticancer potential of the optimized nanoparticles containing AP in rats bearing HCC and could be a future hope for lingering the survival in hepatic cancer patients (Bhattacharya et al., 2018).

### 2.2 Anthocyanidin (ACN)

More than 600 different ACNs have been identified in vegetables, which is derived from twenty-three different aglycones classified according to the number and position of hydroxyl and methoxyl groups on the flavan nucleus. The chromophore of ACNs is the 7-hydroxyflavylium ion. ACNs are responsible for the red, purple and blue colors of many flowers, cereal grains, fruit, and vegetable. Indeed, ACN possess high antioxidant capacity and could play a key role in the prevention of oxidative stress by scavenging ROS and free radicals and modulating endogenous defense system, as demonstrated in several *in vitro* and *in vivo* studies (Galvano et al., 2004; Zafra-Stone et al., 2007).

#### 2.2.1 Effects of ACN on hepatic inflammation

NF-κB and Nrf2 are major transcription factors involved in the regulation of pro-inflammatory and antioxidant genes, respectively. Hwang et al. investigated the antiinflammatory effects of an ACN fraction from purple sweet potato (AF) in dimethylnitrosamine-induced and tert-butyl hydroperoxide-induced liver injury separately and found that AF decreased cyclooxygenase-2 and inducible NO synthase (iNOS) expression by inactivating NF-κB. Their study implied that the ability of AF to mediate NF-κB inhibition may be achieved through the activation of Nrf2, as well as by having a direct antiinflammatory effect (Hwang et al., 2011a; Hwang et al., 2011b).

Most recently, a study reported that Lingonberry (Vaccinium vitis-idaea L.) which is rich in ACN could improve NAFLD by reducing hepatic lipid accumulation, oxidative stress and inflammatory response, and might be mediated through the biosynthesis restoration of Nrf2 and glutathione (Hewage et al., 2021). It has also been documented that a ACN-rich cranberry peel extract ameliorated steatosis and inflammatory signs in the liver, decreasing serum and liver triglyceride contents, ALT and aspartate aminotransferase (AST) activities, as well as diminishing TNFα and TGFβ levels in serum (Zavodnik et al., 2019). Previous studies also demonstrated that ACNs from maize counteracted liver ROS directly, increased superoxidase dismutase 1 (SOD-1) and ameliorated liver damage, indicating that the observed reduction in oxidative stress can be attributed to the ROS scavenging activity of cyanidin-3-O-glucoside (Magaa-Cerino et al., 2020). In NAFLD-induced liver injury, ACN attenuated oxidative stress and inflammation by three independent mechanisms: inhibition of lipogenesis by reducing sterol regulatory element binding protein-1c (SREBP-1c), promotion of lipolysis by induction of PPARα activity, and reduction of oxidative stress (Valenti et al., 2013).

#### 2.2.2 Effects of ACN on liver fibrosis

It has long been thought that TGF-β1, as an important target of hepatic fibrosis, achieved pro-fibrotic effect by TGF-β/Smad signal transduction pathway (Chen and Yang, 2016; Hu et al., 2018). This is supported by Jun et al.‘s work demonstrating that ACN from gooseberry inhibited hepatic fibrosis by blocking the TGF-β/Smad signaling pathway, and reduced the deposition of α-smooth muscle actin (α-SMA) protein and the expression of collagen I (Gao et al., 2020). Meanwhile, ACN from Aronia melanocarpa Elliot could prevent liver fibrosis by inactivating α-SMA expression, reducing the expression of inflammatory factors such as TNF-α and IL-1 and inhibiting the TGF-β1 secretion and Collagen I deposition (Yang et al., 2020). ACN from Eugenia jambolana may reduce the severity of hepatocellular fibrosis and serum ALT levels. More important, their results revealed that ACN can significantly decrease MDA levels which is a biomarker for lipid peroxidation secondary to generation of ROS and reduce inflammation by preventing upregulation of pro-inflammatory cytokines, such as Interleukin 6 (IL-6), Interleukin 1b (IL-1b) and chemokine (C-X-C motif) receptor 2 (Cxcr2) (Donepudi et al., 2011). On the other hand, Cyanidin-3-O-b-glucoside, as a abundant monomer of ACN, could protect against liver fibrosis induced by alcohol via regulating energy homeostasis and adenosine 5′-monophosphate-activated protein kinase (AMPK)/autophagy signaling pathway. These findings strongly suggested that the antifibrotic effects of ACN were significantly depending on inflammation suppression and antioxidative mechanisms (Wan et al., 2017).

#### 2.2.3 Effects of ACN on liver cancer

The anticancer effects of ACN on HCC cells have been extensively studied. ACN in blueberries could inhibit the proliferation of human hepatocellular cancer HepG2 in a dose-dependent manner. Their results of Western blotting showed that the expression of Caspase-3 protein increased significantly in the treatment group, suggesting that ACN could be developed as an agent against liver cancer (Li et al., 2014). In addition, Jie Lin and colleagues systemically evaluated the mechanism driving the anticancer function of Mv-3-gal which is a major monomer in blueberry ACN by focusing on apoptosis and metastases pathways. Their experiments for the first time demonstrated that Mv-3-gal could remarkably induce G1/S phase cell arrest and apoptosis of human HCC cell Huh-7 cells via regulating p38/Jun N-terminal kinase (JNK)/ERK MAPK and protein kinase B (Akt)/phosphatase and tensin homolog (PTEN) signal pathways. Further more, Mv-3-gal could inhibit the ability of migration and invasion of Huh-7 cells, and the expressions of MMP-2 and MMP-9 at mRNA level (Lin et al., 2020). ACN-rich peels of Myrtaceae fruits reduced the serum levels of the liver injury marker ALT. In which, M. jaboticaba peel reduced the incidence of liver preneoplastic foci, hepatocyte proliferation (Ki-67), protein levels of hepato-mitogen TNF-α, diminished liver lipid peroxidation and increased total glutathione levels, indicating a protective effect of M. jaboticaba on preneoplastic lesion development and fibrosis (Romualdo et al., 2020). Another recent study suggested that ACN from purple sweet potato can participate in the process of apoptosis mediated by human HCC cell SNU-387 through an exogenous apoptotic pathway mediated by death receptor TNFR1 and MAPK signaling pathway (Li et al., 2018).

### 2.3 Isoflavone

Isoflavones are a large and distinct subclass of flavonoids, which have a limited distribution in the plant kingdom and are found mainly in soybeans and other legumes, having great potential to fight a wide range of diseases.

#### 2.3.1 Genistein (GEN)

GEN, a soybeanderived isoflavone, is a naturally occurring phytoestrogen with both chemopreventive and chemotherapeutic potential, including inhibition of tumor cell growth (Lee et al., 2009), antihepatic fibrosis (Cao et al., 2017), etc.

##### 2.3.1.1 Effects of GEN on hepatic oxidative stress

It has been revealed that GEN could significantly inhibited the increased protein levels of iNOS and cyclooxygenase-2, thereby reducing nitric oxide (NO) and prostaglandin-E (PGE) levels, respectively. Furthermore, GEN was able to maintain the redox potential and strengthen the antioxidant defense system of a cell (Ganai et al., 2015). GEN could ameliorate hepatic inflammatory reaction in nonalcoholic steatohepatitis (NASH) rats, possibly by reducing TLR4 protein and gene expression, decreasing the endotoxin and TNFα, alleviating the inflammatory reaction and making the indicators detected in blood and liver stay near normal in NASH rats (Yin et al., 2019). Based on the findings of Salahshoor et al., the administration of GEN as an antioxidant and a potent phytoestrogen could reduce the morphine-induced injuries through various mechanisms and improve the structure, enzymes, and performance of liver in the groups receiving morphine (Salahshoor et al., 2018). Furthermore, it has been reported that GEN can prevent and protect against Acetaminophen-induced liver toxicity due to the inhibition of Acetaminophen biotransformation and the resistance to oxidative stress via the modulation of the activities of metabolism and the antioxidant enzyme (Fan et al., 2013). In N-nitrosodiethylamine-induced hepatotoxicity, pretreatment of GEN (25, 50 and 100 mg/mL, orally) markedly relieved genotoxic damage, improved oxidative stress and reduced serum levels of blood serum enzymes (Ali et al., 2015). Excessive oxidative stress-induced depletion of glutathione usually leads to accumulation of ROS and following mitochondrial and cell damage. It is interesting to report that the decrements of AST and ALT and increments of total protein levels (TP), albumin and glutathione in GEN group more than in glucurolatone group in a comparative study on detoxifying and protective effects of GEN and glucurolactone against liver injury which means the antihepatic damage capacity of GEN is better than glucurolatone (Li et al., 2012). Another study showed that GEN improved liver necrosis induced by toxic doses of paracetamol more markedly than SL (Mustika and Achmad, 2014).

##### 2.3.1.2 Effects of GEN on liver fibrosis

GEN has been reported to possess various therapeutic/nutraceutical effects, including fibrosis (Ganai and Farooqi, 2015). GEN employs multiple signaling pathways to exert the inhibitory effects on fibrosis. GEN improved schistosomiasis-induced liver fibrosis associated with strong elevation of sirtuin 1 (SIRT1) expression and activity (Zhou et al., 2021). The key proteins of combination therapy with taurine, epigallocatechin gallate and GEN against liver fibrosis were founded in 2016. This study suggested that thioredoxin 1 is a key target protein in regulating liver fibrogenesis and upregulation of thioredoxin1 may be one of the antifibrotic mechanisms of combination therapy. Moreover, there are credible evidences that GEN produced hepatoprotection through modulating the expression and phosphorylation of epidermal growth factor receptor (EGFR) in experimental fibrosis (Rodriguez and Alvarez, 2016). GEN had an effect on improving acetaminophen (APAP)-induced liver damage and hepatic fibrosis in mice by reducing the expressions of connective tissue growth factor, TNF-α and IL-6 (Song H. et al., 2018). The functional role of TGF-β/Smad signaling in liver fibrosis has been demonstrated by the finding that Smad3 downregulation ameliorated dimethylnitrosamine-induced hepatic fibrosis (Latella et al., 2009). Ganai et al. suggested that induction of hepatic Smad7, thereby inhibiting activation of TGF-β/Smad signaling, may be an underlying mechanism by which GEN protected against chronic liver disease associated with fibrosis (Ganai and Husain, 2017). GEN has positive effects on liver damage and fibrosis by decreasing the levels of TNF-α, PDGF-BB and IL-6, which were elevated by CCl4 (Demiroren et al., 2014).

##### 2.3.1.3 Effects of GEN on liver cancer

GEN has been identified as a potential cause for the low incidence of certain types of tumors, such as breast, gastric, colon, and HCC. This is supported by Wang et al.‘s work demonstrating that MMP-9 inhibitory activity of GEN and its inhibition of multiple signal transduction pathways such as MAPK, inhibitor of NF-κB (IκB), and phosphoinositide 3-kinase (PI3K)/Akt could control the invasiveness and metastatic potential of HCC (Wang et al., 2014). Furthermore, GEN are reported to induce liver cancer MHCC97-L cells apoptosis and activity of caspase-3 and caspase-9 through inhibiting JNK pathway (Liu et al., 2019). In a different study, the authors documented an anti-proliferative and apoptotic effect of GEN and the sensitivity to fluorouracil, using a canonical SMMC-7721 cell line. In this system, GEN (10 μmol/L) could effectively reverse the resistance of the cells to fluorouracil and its mechanism of inhibits characteristics of liver cancer stem cells is likely involved in inhibiting protein expression of stem cell markers and obstructing Hedgehog signal pathway (Li et al., 2015). In recent years, more and more researchers have begun to focus on solubilizing preparations of GEN for its low aqueous solubility and first-pass metabolism. There was a complexation with phospholipids being made which enhanced *in-vivo* antitumor effect compared to GEN suspension on diethylnitrosamine (DEN)-induced HCC in mice (Komeil et al., 2021). Furthermore, the nanoparticles of star-shaped copolymer mannitol-functionalized were developed for GEN delivery for liver cancer treatment. The studies *in vivo* indicated that the GEN-loaded star-shaped copolymer mannitol-functionalized nanoparticles were able to show significantly superior antitumor activity than the linear copolymer mannitol-functionalized nanoparticles and pristine GEN (Wu et al., 2016). These results may support the potentially effective chemopreventive and/or chemo therapeutic of GEN against liver cancer.

#### 2.3.2 PA

A large number of studies have shown that various natural active products of pueraria lobata, especially PA, could play a protective role in different types of liver diseases by regulating oxidative stress, inflammatory response, lipid metabolism, etc.

##### 2.3.2.1 Effects of PA on hepatic oxidative stress

PA has potential antioxidative activity both *in vitro* and *in vivo*. This is supported by Liu et al.‘s work demonstrating that PA could protect the rat liver against lead-induced injury by reducing ROS production, renewing the activities of antioxidant enzymes and decreasing DNA oxidative damage (Liu et al., 2012). Liver is the main target organ of cadmium toxicity. PA could significantly downregulate hepatic MDA and ROS levels in a cadmium-induced liver injury model, demonstrating the antioxidant effect of PA against cadmium exposure (Wan et al., 2021). Likewise, PA restored cadmium-induced Nrf2 inhibition to prevent autophagy inhibition and NLRP3 inflammasome activation, providing novel insights into the protection of PA against cadmium-induced hepatic cell damage (Yu et al., 2021). Excessive use of acetaminophen can cause oxidative stress in the liver and eventually lead to acute liver injury. PA can protect APAP-induced liver injury via alleviating oxidative stress and mitochondrial dysfunction by affecting the nuclear migration of Nrf2 via inhibiting Keap1 (Zhou et al., 2023). PA most likely prevented activation of the pro-inflammatory factors and reduced LPS/D-Gal-induced liver injury by enhancing the zinc finger E-box-binding homeobox 2 expression level, and consequently, blocking activation of the NF-κB signaling pathway in the liver (Yang et al., 2021a).

##### 2.3.2.2 Effects of PA on hepatic lipid metabolism

PA was considered healthy food agents with tremendous potential to alleviate NAFLD. An intervention experiment with PA in a high-fat-diet (HFD)-induced NAFLD mouse model had demonstrated that alterations of the gut microbiota upon PA intervention had a beneficial effect on high-density lipoprotein cholesterol-induced hepatic steatosis, which plays a key role in ameliorating NAFLD (Wang X. et al., 2024). Furthermore, PA could inhibit LPS-producing genus *Helicobacter*, and promote butyrate-producing genus Roseburia which revealed the protective effect of PA against NASH (Gong et al., 2021). In addition, PA ameliorates hepatic steatosis through several mechanisms. In a study of molecular mechanisms in which PA regulated adipogenesis and lipolysis in human HepG2 cells, the hepatic steatosis could be improved by activating G protein-coupled estrogen receptor (GPER) (Pham et al., 2022). Other studies have shown that PA improved hepatic glucose and lipid homeostasis *in vitro* and *in vivo* by regulating the AMPK pathway, which indicated that PA showed potential as a functional food therapeutic for the treatment of obesity (Xu et al., 2021). In mice fed with high-fat and high-fructose diet for 20 weeks, PA can ameliorate hepatic lipid accumulation in NAFLD rats by reducing the expression of genes associated with lipid traits, such as Srebf1, Chrebp, Acaca, Scd1, Fasn, Acacb, Cd36, Fatp5, Degs1, Plin2, and Apob100 and upregulating the expression of Mttp, Cpt1a, and Pnpla2 (Zhou et al., 2022). Meanwhile, PA ameliorated NAFLD by suppressing ferroptosis and inflammation via the SIRT1/Nrf2 signaling pathway (Yang et al., 2023). Many NAFLD patients suffer from severe ischemia-reperfusion injury (IRI). Yang et al. demonstrated that PA could significantly protect the fatty liver against IRI by activating the PI3K/AKT signaling pathway (Yang X. et al., 2024).

##### 2.3.2.3 Effects of PA on alcoholic liver disease (ALD)

ALD is a growing global health concern, and its early pathogenesis includes steatosis and steatohepatitis. Hu et al. found that PA (100 mg·kg-1) attenuated EtOH-induced liver injury and inhibited the levels of SREBP-1c, TNF-α, IL-6 and IL-1β, and mechanistically, PA might suppress liver lipid accumulation and inflammation by regulating MMP8 (Hu et al., 2023). Network pharmacological predictions combined with animal experiments suggested that PA and GEN in Pueraria lobata may increase hepcidin production to alleviate alcohol-induced iron overload by inhibiting the MAPK/ERK signaling pathway (Li et al., 2024). PA could alleviate alcohol-induced hepatic steatosis in zebrafish larvae by regulating alcohol and lipid metabolism, which was closely related to the regulation of the AMPKα-acetyl-CoA carboxylase (ACC) signaling pathway (Liu et al., 2021). Another study showed that, PA-loaded mesoporous silicon nanoparticles through mammalian target of rapamycin (mTOR)-mediated autophagy pathway could be a possible protective strategy to improve alcoholic hepatitis (Zhang X. X. et al., 2022). There is a research demonstrating that the possible cytoprotective mechanisms of PA may be involved inhibition of the cyclooxygenase-2 pathway and the 5-lipoxygenase pathway to suppress inflammatory response and regulate the protective factor PPAR-γ expression in a chronic alcoholic liver damage model (Tian et al., 2021).

##### 2.3.2.4 Effects of PA on liver fibrosis

It has long been thought that PA was effective in the treatment of chemical-induced liver fibrosis in rats and the primary mechanisms of this therapeutic effect could be due to its protection against hepatic injury by reducing serum levels of ALT and AST, as well as its induction of apoptosis in activated HSC by downregulating bcl-2 mRNA expression (Zhang Y. et al., 2006). Furthermore, PA successfully reversed hepatotoxicity in CCl4-induced hepatic fibrosis rats via the underlying mechanisms of regulating serum enzymes and attenuating TNF-α/NF-κB pathway for anti-inflammation response, as well as improving metabolic function in liver tissue, which might be one of the regulator mechanisms of antifibrosis (Li et al., 2013). PA played a protective role in CCl4-induced liver fibrosis probably through inhibition of PARP-1 and following reduction of NF-κB, ROS production and mitochondrial dysfunction (Wang et al., 2016). Another study identified a special effect for PA-regulation of the TGF-1/Smad pathway in fibrotic development and provide a promising program for hepatofibrosis treatment (Xu et al., 2013).

##### 2.3.2.5 Effects of PA on liver cancer

Epithelial-mesenchymal transition (EMT) is an essential step during the progression of metastasis in the vast majority of cancers. PA improved the mesenchymal characteristics through the TGF-β1/Smad3 pathway (Wu et al., 2018). This is supported by Zhou et al.‘s work demonstrating that PA affected HCC through the miR-21/PTEN/EMT regulatory axis (Zhou et al., 2020). The combined therapy may present a novel therapeutic method for HCC. There is a research demonstrating that a marked increase in the inhibition of tumor growth and the number of apoptotic cells in response to combined treatment with PA and 5-fluorouracil was identified with no observed liver or renal toxicity (Zeng et al., 2014). Overall, PA may represent a chemopreventive agent for HCC treatment.

### 2.4 Chalcone

The majority of naturally occurring chalcones are polyhydroxylated aromatic compounds abundantly found in fruits, grains, legumes, vegetables, and beverages such as tea, coffee, red wine, beer, etc. Chalcones are known to exhibit diverse therapeutic and pharmacological activities such as antioxidant and antiinflammatory.

#### 2.4.1 Isoliquiritigenin (ISL)

ISL, a flavonoid with a chalcone structure, is a bioactive ingredient isolated from the roots of plants belonging to licorice that exhibits antioxidant, antiinflammatory, and phytoestrogenic activities.

##### 2.4.1.1 Effects of ISL on hepatic oxidative stress

ISL pretreatment prevented from triptolide-induced hepatotoxicity by inhibiting MDA and restoring the levels of glutathione, glutathione peroxidase (GPX), SOD and catalase (CAT) via activation of the Nrf2 pathway in an acute liver injury model (Cao et al., 2016). It has been reported that ISL improved the ability of antioxidative stress, alleviated inflammatory reaction, apoptosis, and inhibited NLRP3 inflammasome to protect LPS/D-GalN-induced acute liver failure through activating the PPAR-gamma coactivator (PGC)-1α/Nrf2 pathway (Wang X. et al., 2021). Consistently, ISL attenuated emodin-induced hepatotoxicity *in vivo* and *in vitro* through Nrf2 pathway (Ni et al., 2022). Besides, ISL treatment not only effectively reduced TNF-α and IL-6 gene expression in LPS-induced mouse primary peritoneal macrophages but also attenuated lung and liver injuries induced by LPS, accompanied by decreased inflammatory responses, which may be strongly related to NF-κB inhibition (Chen et al., 2018). There is a research demonstrating that an optimized ISL derivative functionally blocked TLR2/MyD88/NF-κB signaling pathway to downregulate the production of inflammatory cytokines resulted significant liver protection property (Yang et al., 2021b). A recent study also showed that ISL alleviated doxorubicin-induced hepatocyte toxicity by exciting the Nrf2/antioxidants axis and following inhibition of NF-κB, mainly by upregulating SIRT1 (Al-Qahtani et al., 2022).

##### 2.4.1.2 Effects of ISL on hepatic lipid metabolism

Ethanol can dysregulate lipid metabolism and cause hepatocyte injury. It has shown that ISL as a potent agonist of SIRT1-AMPK signaling in ethanol exposure-induced steatosis following prevent ethanol-induced lipid accumulation through not increasing fatty acid oxidation but blocking lipid synthesis (Na et al., 2018). ISL could alleviate ALD, and further verified that ISL exerted protective effects through modulating miR-23a-3p/PGC-1α-mediated lipid metabolism *in vivo* and *in vitro* (Wang et al., 2022a). There are studies suggesting that ISL could improve NAFLD through multiple pathways. This is supported by Hu et al.‘s work demonstrating that ISL could alleviate methionine-choline-deficien (MCD) -induced NAFLD in mice involved with the inhibition of macrophage inflammatory activity by the blockade of Syk-induced inflammasome activation (Hu et al., 2024). There is a research finding that ISL alleviated NAFLD through modulating miR-138-5p/PGC-1α-mediated lipid metabolism and inflammatory reaction *in vivo* and *in vitro* (Wang et al., 2022b). More important, Zhang et al. observed that ISL mitigated NAFLD/NASH symptoms by downregulating SREBP and upregulating PPARα activities via the IQGAP2-CREB-SIRT1 axis (Zhang et al., 2021).

##### 2.4.1.3 Effects of ISL on liver fibrosis

The therapeutic role of ISL in liver fibrosis gaining prominence. In a recent study, zebrafish, HSC-T6 cells, and mice were used as the research object. They first elucidated that ISL relieved liver fibrosis by inducing HSCs ferroptosis through repressing GPX4 expression and increasing the expression of transferrin receptor (TFR) and divalent metal transporter 1 (DMT1), thus producing a large number of ROS (Huang et al., 2022). Furthermore, ISL improved the area of liver pathological stasis and deposition of fibrillar collagen and inhibited the mRNA expression levels of IL-6, TNF-α and IL-1β in liver tissues in an animal model of alcoholic liver fibrosis. And it was found for the first time that ISL could inhibit Annexin A2 expression both *in vivo* and *in vitro*, block the sphingosine kinases/sphingosine-1-phosphate/IL-17 signaling pathway and regulate the expression of α-SMA by inhibiting the phosphorylation of STAT3 at the downstream signal to finally reverse HSCs activation and hepatic fibrosis (Liu et al., 2023).

##### 2.4.1.4 Effects of ISL on liver cancer

The known anticancer mechanisms of ISL involve inhibiting cell proliferation, inducing cell apoptosis and cycle arrest, and promoting oxidative stress. There is a research first demonstrating that ISL suppressed the cell cycle transition and inhibited the proliferation and migration of Hep3B cells *in vivo* and *in vitro* by suppressing cyclin D1 and PI3K/AKT pathway (Huang et al., 2020). Consistently, ISL induced apoptosis and autophagy through inhibition of the PI3K/Akt/mTOR pathway in HCC MHCC97-H and SMMC7721 cells (Song et al., 2020). In addition, ISL induced HepG2 cell apoptosis via ROS-mediated MAPK, STAT3, and NF-κB signaling pathways (Wang et al., 2019). Licorice flavonoids showed strong inhibitory effects on Bel-7402 cancer cells, among which ISL exhibited the greatest potency against Bel-7402 (IC50 = 0.7 ± 0.11 mol/L), which was 3-fold more potent than flavopiridol (2.4 ± 0.34) mol/L in an antitumor activity of licorice flavonoids (Zhang and Li, 2018).

#### 2.4.2 Hydroxysafflor yellow A (HSYA)

HSYA extracted from the flower of Carthamus tinctorius L., as a chalcone glycoside, can effectively increase the activities of antioxidant enzyme and reduce oxidative stress mediated damage, which was extensively used in traditional Chinese medicine to treat cirrhosis. It has been shown that HSYA was able to significantly protect the liver from oxidative stress by increasing the activities of antioxidant enzymes, upregulating the expression of PPAR-γ and MMP-2, and downregulating the expression of TGF-β1 and tissue inhibitor of metalloproteinases-1 (TIMP-1), and reducing α-SMA level, which indicating that PPARγ-TGF-β1 would be a signal pathways for HSYA to reduce liver fibrosis induced by oxidative stress (Wang et al., 2013). HSYA attenuated CCl4-induced liver injury and blocked TGF-β1-regulated HSC activation by downregulating α-SMA, collagen α type I, MMP-9, and TIMP-1 gene expression (Zhang et al., 2011). In chronic CCl4/HFD-induced liver injury, HSYA ameliorated serum biochemical indicator, reduced mRNA expression levels of pro-fibrotic factors and elevated hepatoprotective factors, which revealing that PPAR-γ and p38 MAPK signaling play pivotal roles in the prevention of liver fibrosis (Liu et al., 2014). In addition, it has been reported that HSYA can inhibit the proliferation and migration abilities of HepG2, Hep3B and SMMC7721 cells, and induce the apoptosis of HepG2 cells by blocking PI3K/Akt signaling pathway (Song L. et al., 2018).

### 2.5 Dihydroflavone

Dihydroflavones are usually found in all citrus fruits such as oranges, lemons and grapes. Hesperidin and Naringenin are typical dihydroflavones compounds. There are many significant pharmacological effects as antioxidants, antiinflammatory, hypolipidemic and hypocholesterolemic, due to their free radical scavenging properties.

#### 2.5.1 Hesperidin (HD)

##### 2.5.1.1 Effects of HD on hepatic oxidative stress and inflammation

The antioxidative effects of HD have been verified in numerous chemical-induced liver injury animal models. HD has been reported to alleviate Diazinon-induced hepatotoxicity via antioxidant and antiinflammatory properties (Mesallam and Atef, 2020). Additionally, HD has been shown to protect against Valproate-induced hepatotoxicity by its scavenging of free radicals, antioxidant property and prevent oxidative stress mediated apoptosis, as assessed by liver marker enzymes, oxidative stressors, antioxidant enzymes, mRNA and protein expression of apoptotic proteins (Nathiya et al., 2016). A previous study has shown that HD could effectively play a role in fighting against immunological liver injury induced by concanavalin A through the reduction serum levels of ALT and AST, the content of MDA in liver homogenates and the hepatic caspase-3 activity and TNF-α level (Suresh et al., 2014). A later study has also confirmed the antioxidant and chemoprotective abilities of HD in liver tissues through the reduction serum levels of ALT, AST, triglyceride and total cholesterol levels, liver MDA and NO content and elevation of glutathione content in cisplatin-induced rats model (Omar et al., 2016). It is interesting to report that a derivative from HD which was modified enzymatically through glycosylation and de-rhamnosylation could alleviate ALD through preventing excessive lipid formation, protecting the antioxidant system and suppressing induction of inflammation in hepatocytes (Park et al., 2013). An orange peel extract in which HD is the main compound can protect rat liver from CCl4-induced injury by lowering the levels of AST and ALT, and by increasing the content of glutathione, the activities of SOD and glutathione peroxidase (Chen et al., 2013).

##### 2.5.1.2 Effects of HD on hepatic lipid metabolism

HD has been reported to have a therapeutic effect on lipid disorders in the liver. Emerging evidence indicated that HD may improve hypercholesterolemia and fatty liver by inhibiting both the synthesis and absorption of cholesterol and regulating the expression of mRNA for retinol binding protein, cutaneous and heart fatty acid–binding protein (Wang et al., 2011). It has been reported that HD attenuates lipid accumulation *in vivo* and *in vitro* via AMPK activation, as shown in HD increased the expression level of pAMPK and downregulated SREBP-1C, ACC and fatty acid synthase expression (Chen et al., 2022). Previous studies have shown that HD improved NAFLD by promoting fatty acid β-oxidation through activating SIRT1/PGC1α in HFD models *in vitro* and *in vivo* (Nie et al., 2024). As reported, HD supplementation, was associated with a significant reduction in ALT, γ-glutamyltransferase, total cholesterol, triglyceride, hepatic steatosis, TNF-α, and NF-κB in a randomized, placebo-controlled, double-blind clinical trial (Cheraghpour et al., 2019). Additionally, HD dramatically reduced the hepatic morphological damage and the expressions of alcohol and lipid metabolism related genes, including cyp2y3, cyp3a65, hmgcra, hmgcrb, fasn, and fads2 in an ALD zebrafish larvae model induced by 350 mM ethanol for 32 h (Zhou Z. et al., 2017).

##### 2.5.1.3 Effects of HD on liver fibrosis

The antifibrosis effect of HD may be associated with its free-radical scavenging ability. It has been documented that HD (50, 100, 200 mg/kg) not only reduced the levels of ALT, AST, hyaluronic acid, laminin, procollagen III n-terminal peptide, collagen IV, MDA, TGF-β1 significantly and the degree of hepatic fibrosis, but also increased SOD activity in a CCl4-induced liver fibrosis model in rats (Wu et al., 2011). In a similar experimental model, HD significantly decreased hepatic TGFβ1, hydroxyproline, the serum liver function markers of ALT, AST and total bilirubin, the hepatic content of MDA and myeloperoxidase activity, the serum pro-inflammatory cytokine TNF-α, relative liver weight, and the serum lipid profile markers cholesterol, triglycerides and low-density lipoprotein compared with the reference drug SL, which suggested that HD may be a promising protective agent against liver fibrosis (Abdel-Sttar et al., 2017). Evidence also suggested that HD has significant positive effects on liver morphology and structure, inflammation, fibrosis, and oxidative stress in rats with bile duct ligation (BDL)-induced cholestatic liver injury by decreasing the levels of inflammatory gene expression and increasing the levels of total antioxidant capacity (Nasehi et al., 2023). A HD derivative was found to suppress HSC activation and proliferation by targeting PTEN/AKT pathway, which means it is a potential therapeutic choice in treating liver fibrosis (Li et al., 2017).

##### 2.5.1.4 Effects of HD on liver cancer

The anticancer effects of HD and its derivatives have been extensively studied. It has been reported that HD induced paraptosis like cell death in HepG2 cells through the phosphorylation of ERK1/2 (Yumnam et al., 2014). Recently, researchers found that HD showed antioxidant, antiinflammatory and proapoptotic activities by significantly prevented thioacetamide-activated Wnt3a/β-catenin and Wnt5a pathways in thioacetamide-induced early HCC model in rats, which suggested that HD exhibited an antitumor and hepatoprotective effects against the development of HCC (Zaghloul et al., 2017). Hasanin et al. reported that HD could suppress liver carcinogenesis in rat model by decreasing exosomal RAB11A messsenger RNA and long noncoding RNA-RP11-583F2.2 along with the increase in exosomal miR-1298, involved in the exophagy process (Hasanin et al., 2020).

#### 2.5.2 Naringenin (NIN)

##### 2.5.2.1 Effects of NIN on hepatic oxidative stress

Recently, the hepatoprotective effects of NIN in the chemicals- and toxicant-induced liver injury have been investigated. NIN could provide antiinflammatory and antioxidant protection for Dasatinib-induced liver damage by minimizing the proinflammatory mediators, such as IL-10, TNF-α and MDA, whereas restoring the levels of antioxidant genes including SOD, CAT, and glutathione-s-transferase (GST) (Alanazi et al., 2023). Emerging evidence suggested that NIN diminished the oxidative stress and inflammation induced by doxorubicin through reducing inflammatory molecules and replenishing reduced antioxidant armory (Wali et al., 2020). NIN also significantly prevented APAP-induced liver lesions, liver adipose lesions, hepatocyte injury and apoptosis by reducing the oxidative stress damage of mouse liver cells and the inflammation-related factors in mice model (Xu et al., 2024). Similarly, the pretreatment with NIN (10 mg/kg) significantly increased the level of serum albumin, reduced high hepatic lipid peroxidation and increased liver glutathione content as well as the activities of superoxide dismutase and glutathione peroxidase in paclitaxel-induced liver injury model (Khaled et al., 2023). Additionally, a novel NIN-loaded nanoparticles system was developed to resolve the restricted bioavailability of NIN, which could reduced liver function index and lipid peroxidation, in conjunction to a substantial increase in the levels of the antioxidant enzymes (Yen et al., 2009).

##### 2.5.2.2 Effects of NIN on hepatic lipid metabolism

As a famous energy sensor and regulator, AMPK plays a key role in energy expenditure (Shrikanth and Nandini, 2020). A previous study has shown that NIN alleviated NAFLD by increasing energy expenditure and regulating autophagy via activating AMPK (Yang Y. et al., 2021). Nevertheless, the direct binding of NIN and AMPKγ1 needs further validation. It has also been indicated that NIN prevented NAFLD via downregulating the NLRP3/NF-κB signalling pathway both in Kupffer cells and in hepatocytes, thus diminishing inflammation in the mice livers (Wang X. et al., 2020). In addition, a clinical study reported that NIN administration may be useful for treating NAFLD by modulating energy balance, glucose and lipid metabolism, oxidative stress, and inflammation through different mechanisms (Naeini et al., 2021). The balance between gut microbiome and host metabolome has become recognized as important in the maintenance of physiological function. It is noteworthy that NIN could prevent nonalcoholic steatohepatitis by modulating the host metabolome and intestinal microbiome in MCD diet-fed mice (Cao et al., 2023a).

##### 2.5.2.3 Effects of NIN on liver fibrosis

A previous study has demonstrated that NIN ameliorated liver injury and liver fibrosis, decreased collagen deposition and cyclic guanosine monophosphate-adenosine monophosphate synthase (cGAS) expression, and inhibited in flammation in CCl4-treated mice, which indicating that interrupting the cGAS-stimulator of interferon genes pathway contributed to reverse the fibrosis process (Chen H. et al., 2023). Another study in 2019 indicated that NIN reduced α-SMA and Smad3 protein and mRNA levels and blocked oxidative stress, inflammation and the TGF-β-Smad3 and JNK-Smad3 pathways, thereby carrying out its antifibrotic effects (Hernández-Aquino et al., 2019). A recent study has further demonstrated that NIN administration markedly alleviated liver injury and fibrosis by significantly increasing autophagic activity in HSCs, concurrently inducing ferroptosis in mice subjected to BDL (Yu et al., 2024).

##### 2.5.2.4 Other hepatoprotective effects of NIN

Goldwasser et al. found that NIN dose-dependently inhibited hepatitis C virus (HCV) without affecting the intracellular levels of the viral RNA or protein and blocked the assembly of intracellular infectious viral particles, upstream of viral egress (Goldwasser et al., 2011). The NIN nanoparticles were prepared using nanoprecipitation method could inhibit HepG2 cells proliferation (Elnawasany et al., 2023).

### 2.6 Dihydroflavonol

#### 2.6.1 SL

About 50%–70% of the silymarin extract consists of the flavonolignan SL, which is extensively studied and regarded as the most active component of silymarin (Loguercio and Festi, 2011). SL has been used for centuries in medicine, mainly to treat kidney, spleen, liver, and gallbladder diseases, such as hepatitis and cirrhosis (Flora et al., 1998; Schadewaldt, 1969). It also protects the liver from various chemical and environmental toxins, such as snake bites, insect bites, poisonous mushrooms, alcohol and acetaminophen (Kren and Walterova, 2005; Saller et al., 2001; Mashhadi Akbar Boojar et al., 2016; Ramasamy and Agarwal, 2008). Nowadays, 65% of the patients with liver diseases take herbal preparations, which are mainly derived from SL (Loguercio et al., 2012).

##### 2.6.1.1 Effects of SL on inflammatory

Emerging evidence suggested that SL could fight inflammation through the suppression of NF-κB signaling pathway and activation of TNF-α, attenuate autoimmune, allergic, preeclampsia, cancer, and immune-mediated liver diseases and suppress oxidative and nitrosative immunotoxicity (Esmaeil et al., 2017; Milić et al., 2013). There is a result showing that SL could improve oxidative and nitrosative stress and inflammation-mediated tissue damage by preventing the activation of pro-inflammatory transcription factors such as NF-κB and STAT-1 (Hou et al., 2010). Hepatic inflammation is evaluated by the extent of lymphocyte infiltration and production of inflammatory cytokines and chemokines, which are often induced by transcription factor NF-κB (Mihm et al., 2004; Polyak et al., 2001; Falasca et al., 2006; Wu et al., 2007). SL could inhibit immune-mediated liver disease in a mouse model of T-dependent hepatitis, induced by intravenous injection of concanavalin A. SL significantly inhibited the intrahepatic synthesis of several disease-related pro-inflammatory cytokine mRNAs, i.e., for TNF, interferon-gamma (IFN-g), IL-4, and IL-2, intrahepatic induction of iNOS, and intrahepatic activation of NF-κB (Morishima et al., 2010; Schumann et al., 2003). SL inhibited colocalization of NLRP3 and apoptosis-associated speck-like protein containing a caspase-recruitment domain (ASC) as well as α-tubulin and acetylated-α-tubulin in response to palmitate treatment, explaining that the NAD+/SIRT2 pathway was an important mediator through which SL prevented NLRP3 inflammasome activation during NAFLD (Zhang et al., 2018). In conclusion, SL could play a related role in its hepatoprotective effect. The potential mechanisms include inhibition of the NF-κB pathway, suppression of TNFα, leukotrienes and NO, inhibition of adhesion molecules, and interference with the NAD^+^/SIRT2 pathway, and as an immunomodulator, SL attenuates the function of T lymphocytes.

##### 2.6.1.2 Effects of SL on hepatic oxidative stress

In recent years, there has been an increasing use of SL in the treatment of several other pathologies. This increase can be attributed to its antioxidant capacity as well as its ability to modulate cellular signaling pathways. SL could inhibit oxidative stress, prevent the activation of HSCs and activate immune cells (Kupffer cells), thus reducing oxidative damage, in the presence of elevated liver enzymes. The generation of ROS is a natural consequence of various basic biochemical reactions in the liver and is mainly related to detoxification processes. SL acts as a scavenger of ROS, augments the level of glutathione in the liver, and inhibits hepatic NF-κB activation (Gillessen and Schmidt, 2020). SL inhibited lipid peroxidation and the formation of free radicals, and stimulated the synthesis of proteins and phospholipids in hepatocytes. These actions, in turn, protected cells from damage, stabilized the cell membrane, and reduced membrane permeability (Lazebnik et al., 2020). There are many studies on the antioxidant effects of SL, and in general, most of the hepatoprotective properties of SL are attributed to these antioxidant activities.

##### 2.6.1.3 Effects of SL on toxin blockade

SL is a suitable candidate to treat drug-induced and toxic liver injury. SL prevented mitochondrial ROS production, cardiolip inoxidation, and citrate carrier failure in cirrhotic livers but did not affect the expression of lipogenic enzymes. It exerts a regulatory action on cellular and mitochondrial membrane permeability in association with an increase in membrane stability against the xenobiotic injury (Serviddio et al., 2014). The capacity of SL to limit mitochondrial failure is part of its hepatoprotective property. Some researchers have identified the main efflux transport proteins of SL was multidrug resistance-associated protein (MRP2) and breast cancer resistance protein (BCRP), and compounds that inhibited the efflux transport proteins MRP2 and BCRP could enhance the absorption and activity of SL (Xie et al., 2019), such as piperine or tangeretin (Yuan et al., 2018). SL showed weak-to-moderate inhibition of most cytochrome P450 (CYP) enzymes *in vitro* experiments (Xie et al., 2019). Due to the low bioavailability of SL, its concentration in humans rarely reaches a level that constitutes significant inhibition (Gurley et al., 2012). SL inhibited CYP1A2, 2B6, 2C8, 2C9, 2C19, 2D6 and 3A4, but the most prominent inhibitory effects were on CYP3A4 and CYP2C9 (Beckmann-Knopp et al., 2000).

Previous studies have shown that SL protected against various types of drug-induced liver injury. As a hepatoprotective agent, it is frequently prescribed as an adjuvant therapy along with other medications such as methotrexate, which has significant liver toxicity (Hussain and Marouf, 2013). SL played an important protective role in the early stage of acetaminophen-induced acute liver injury by decreasing the activity and expression CYP2E1, reducing the production of toxic metabolites, and alleviating liver injury (Yang et al., 2022). It should be also noted that the antigenotoxic effects of SL, as it prevented Benzo [a]pyrene-7,8-dihydrodiol-9,10-epoxide-DNA adduct formation by modulating the Nrf2 and PXR signaling pathways. In this study, treatment of HepG2 cells with SL modulated detoxification enzymes through Nrf2 and PXR to eliminate Benzo [a]pyrene metabolites which is a group 1 carcinogen accelerating metastasis and angiogenesis (Jee et al., 2020). Recent studies supported that SL significantly reduced ALT and AST levels in patients with tuberculosis who were taking antituberculosis drugs consists of isoniazid, rifampicin, pyrazinamide, and ethambutol (Tao et al., 2019).

##### 2.6.1.4 Effects of SL on viral hepatitis

SL has been reported to possess potent antiviral activities against a number of viruses (e.g., dengue virus, influenza virus, human immunodeficiency virus, and hepatitis viruses) by targeting multiple steps of the viral life cycle. The effect of SL on HCV has been extensively studied, and the antiviral activity of this drug against HCV *in vitro* is well documented. Polyak et al. demonstrated that SL not only inhibited the infection of genotype 2a HCV strain JFH-1, but also blocked the transcription of TNF-α in peripheral blood mononuclear cells and HCC Huh-7 cells as early as 2007. Further mechanistic studies showed that the antiviral effect of SL was achieved by enhancing the JAK-STAT antiviral signaling pathway, which, in turn, inhibited HCV replication (Polyak et al., 2007). Two other studies in 2013 respectively showed that SL could produce antiviral effects against HCV by blocking lectin-mediated endocytosis and impeding HCV infection through targeting the HCV NS4B protein, which is known to mediate the formation of the membrane network where HCV RNA replication occurs (Blaising et al., 2013; Esser-Nobis et al., 2013). From the study analyzed by Wei et al., it was deduced that SL and other antivirals (lamivudine and interferon) have similar efficacy in normalizing AST and an equivalent negative conversion rate of serum HBsAg (Wei et al., 2013).

##### 2.6.1.5 Effects of SL on liver fibrosis

A team of Chinese researchers reported that SL inhibited HSC activation and hepatic apoptosis and prevented the formation of mallory-denk bodies in the NASH liver when SL was orally administered to the male C57BL/6 mice which were fed a MCD diet for 8 weeks, and the effect was related to alteration of lipid metabolism-related gene expression, activation of the Nrf2 pathway and inhibition of the NF-κB signaling pathway in the NASH liver (Ou et al., 2018). El-Lakkany reported that SL could be a convenient and promising therapeutic agent in the treatment of schistosomal liver fibrosis by downregulating content of hydroxyproline, serum levels and hepatic expression of TGF-b1 and MMP-2 and the number of mast cells (El-Lakkany et al., 2012). In a series of CCl4-induced liver fibrosis studies, the oral administration of SL may improve hepatic fibrosis induced by CCl4 in Wistar rats via the decreasing in fibrotic parameters, including connective tissue growth factor (Clichici et al., 2015; Clichici et al., 2016). Moreover, SL improved diet-induced NASH with liver fibrosis by disturbing the role of the inflammatory cytokine and suppressing the activation of HSCs (Kim et al., 2012). The combined application of SL with other drugs, such as caffeine, lisinopril, and taurine, also showed obviously antifibrotic effects through the downregulation of lysophosphatidic acid receptor 1 (LPAR1) and NF-κB expression (Saber et al., 2018; Eraky et al., 2018). The combination of SL and ginger had protective liver action and reduced the severity and incidence of liver fibrosis by increasing apoptotic pathways through elevating caspase activity (Okda et al., 2019).

##### 2.6.1.6 Effects of SL on liver cancer

SL has been a major inducer of apoptosis and used alone or in combination with other anti-tumour drugs to treat many tumors in the last two decades, including skin, breast, lung, colon, bladder, prostate, kidney and liver (Bijak, 2017). Many studies have shown the mechanisms and pathways involved in the anticancer effects of SL. The induction of tumor suppressor agents that act and stimulate apoptosis, such as initiator caspases (8 and 9), and effector caspase (3), p38, p53 and JNK1/2, and suppression of the expression or activity of apoptosis inhibitors such as Survivin and Bcl-2 have been discussed in various cellular studies (Agarwal et al., 2013). The activation of some transcription factors has been induced by UVB, such as E2F1 and protein-1, which is responsible for the transformation of healthy cells into cancer cells. Inhibition of gene expression or receptors for growth factors is another mechanism by which SL inhibits the proliferation of tumour cells. SL has been shown to act as a protective barrier in preventing tumourigenesis by reducing the expression of these factors (Zhu et al., 2016). SL is an inhibitor of various hepatocellular tumours by inducing the pathway of Kip1/p27 messaging and inhibition of cyclins D1, D3 and E as well as cyclin-dependent kinases. The administration of SL showed anticarcinogenic, antimetastatic and apoptotic effects in a dose-dependent manner on HepG2 cells through the Slit-2/Robo-1 pathway (Aykanat et al., 2020). Meanwhile, SL exerted anti-HCC effects through multiple pathways, including suppression of Ki-67 expression and HGF/cMet, Wnt/β-catenin, and PI3K/Akt/mTOR pathways and enhancement of antioxidant defense mechanisms (Yassin et al., 2022). Furthermore, SL has great promise in cancer treatment as an adjuvant therapy used in conjunction with other chemotherapy drugs.

### 2.7 Flavonol

Flavonols are found in a variety of fruits and vegetables, such as onions, kale, lettuce, tomatoes, apples, grapes and berries. A few studies have linked flavonol intake to a wide range of health benefits, including antioxidant potential and reduced risk of vascular disease. Among them, QE are the most widely used.

#### 2.7.1 QE

QE (3,3′,4′,5,7-pentahydroxyflavone), a flavonoid, is widely found in fruits and vegetables and exerts broad-spectrum pharmacological effects in the liver.

##### 2.7.1.1 Effects of QE on hepatic oxidative stress

QE exerts its hepatoprotective effects mainly through its powerful antioxidant capacity, which has been demonstrated in numerous drug-induced liver injury animal models. A study by Çomaklı examined the protective effects of QE on hepatic oxidative stress. They demonstrated that QE could alleviate the harmful effects of vincristine via activation of Nrf2/HO-1 and SIRT1/PGC-1α pathways, and attenuation of oxidative stress, apoptosis, autophagy, and NF-κB/STAT3 pathways (Çomaklı et al., 2023). Jin et al. have shown that QE attenuated toosendanin-induced hepatotoxicity by inducing the Nrf2/GCL/glutathione antioxidant signaling pathway (Jin et al., 2019). It has been reported that QE ameliorated the increased levels of biochemical markers of the liver, attenuated the Ritonavir induced Bax, caspase-3, NF-κB, and eNOS activation and persuaded the Bcl2 and pAkt level, suggesting that QE exerted a hepatoprotective effect through modulating the oxidative stress, inflammation, and apoptosis (Azmi et al., 2020). Another study in 2020 indicated that QE exhibited protective effects against isoniazid-induced liver damage via inhibiting the activation of NLRP3 inflammasome and apoptosis in a SIRT-dependent manner (Zhang et al., 2020). Antioxidative effects of QE were further supported by a recent study showing that QE-loaded nanoliposomes pretreatment activated the SIRT1/Nrf2/NF-κB signaling pathway to improve antioxidant and antiinflammatory effects in amoxicillin/clavulanate-treated rats (Abd El-Emam et al., 2023). Moreover, the administration of QE could decrease the levels of serum triiodothyronine and thyroxine, activity of oxidative stress markers with a parallel increase in antioxidant markers and Nrf2 L thyroxin-induced hyperthyroidism rats (Zhao et al., 2020).

##### 2.7.1.2 Effects of QE on hepatic lipid metabolism

It has been reported that QE alleviated HFD-induced histopathological changes, disorders of lipid metabolism and mitochondrial damage by enhancing frataxin-mediated PINK1/Parkin-dependent mitophagy and highlighting a promising preventive strategy and mechanism for NAFLD by QE (Liu et al., 2018). Consistent with this study, Cao et al. found that QE prevented NAFLD through AMPK-mediated mitophagy and suggested that promoting mitophagy via an upregulation of AMPK might be a promising therapeutic strategy against NAFLD (Cao et al., 2023b). Jiang et al. found that QE remarkably alleviated lipid droplet accumulation and lipid peroxidation by reducing mitochondrial ROS -mediated ferroptosis in steatotic L-02 cells by using a MtROS scavenger (Mito-TEMPO) and ferroptosis specific inhibitor (Fer-1) (Jiang et al., 2022). Oral administration of QE (0.05%) improved MCD-induced NAFLD through attenuating gene expression deregulation, at least in part through PI3K/AKT pathway inactivation (Pisonero Vaquero et al., 2015). It has been well established that lipophagy-related Rab7 has been presumed as a crucial regulator in the progression of ALD despite elusive mechanisms. A previous study demonstrated that QE attenuated hepatic steatosis by normalizing ethanol-imposed Rab7 turnover disorders and subsequent lipophagy disturbances (Lin et al., 2022). Most recently, A study reported that the combination of metformin and QE can reduce hepatic steatosis in palmitic acid-induced HepG2 cells by stimulating autophagy through the cAMP/AMPK/SIRT1 pathway and diminishing inflammatory cytokines (Afshari et al., 2023). It has also been documented that the combined use of QE with rosuvastatin reduced the intensity of oxidative stress and enhanced antioxidant protection activity, resulting in a decrease in apoptosis of hepatocytes (cytokeratin-18 level was 1.27 times decreased), and prevented the development and progression of metabolic disorders associated with NAFLD (Kravchenko et al., 2024).

##### 2.7.1.3 Effects of QE on ALD

Li et al. demonstrated that QE reversed the inhibition of transcription factor EB (TFEB) nuclear translocation incited by ethanol and exhibited similar effect to Torin 1 mTOR activity inhibitor) which could promote TFEB nuclear translocation, indicating that QE could alleviate chronic ethanol consumption induced liver injury and autophagic flux suppression by regulating mTOR-TFEB pathway (Li et al., 2019). Iron deposition is frequently observed in ALD, which indicates a potential role of ferroptosis in its development. Lin et al. reported that QE ameliorated ethanol-induced liver injury by inhibiting ferroptosis via modulating protein kinase RNA-like endoplasmic reticulum kinase (PERK)-dependent mitochondria-associated endoplasmic reticulum membranes formation (Lin et al., 2024). Autophagy and exosomes were individually confirmed to be involved in the pathogenesis of ALD. Liu et al. suggested that QE coordinately activated autophagy and combated exosomes release by restoring lysosome function, and further mitigated ethanol-induced liver damage (Chen L. et al., 2023).

##### 2.7.1.4 Effects of QE on liver fibrosis

In an experiment conducted by Hernandez et al., treatment with QE prevented liver injury in an animal model of CCl_4_-induced cirrhosis by increasing CB2 expression, reducing CB1 expression, promoting apoptosis of HSC and decreasing the number of activated HSCs (Hernandez Ortega et al., 2014). Studies have observed that QE showed hepatoprotective and antifibrotic effects in animal models of liver fibrosis, which may be involved in modulating the HMGB1-TLR2/4-NF-κB signaling pathways (Li et al., 2016). QE also decreased TGF-β levels and HSC activation and promoted degradation of the extracellular matrix by increasing metalloproteinases (Casas-Grajales et al., 2017). Moreover, the antifibrotic mechanisms of QE might be associated with its ability to regulate NF-κB/IκBα, p38 MAPK antiinflammation signaling pathways, and Bcl-2/Bax antiapoptosis signaling pathway to prevent liver cell apoptosis (Wang et al., 2017). Another study suggested that combined QE and simvastatin attenuated hepatic fibrosis in rats by lowering serum levels of GOT, GPT, ALP, decreasing hepatic mRNA levels of sphingosine kinases type 1 (SphK1) and NLRP3 and elevating total antioxidant capacity, glutathione and Nrf2 expression levels, suggesting that targeting the SphK1/NLRP3 pathway could be a prospective therapeutic strategy against liver fibrosis (Salama et al., 2024).

##### 2.7.1.5 Effects of QE on liver cancer

Previous studies have identified that the anticancer effects of QE are prominent. It has been reported that the incubation of HepG2 cells for 24 h with 60 μM QE resulted in numerous cells that had smaller nuclei with chromatin condensation and perinuclear apoptotic bodies. Furthermore, 60 μM QE could induce the poly (ADP-ribose) polymerase as a DNA repair enzyme cleavage in a time-dependent manner (Huang et al., 2013). It has been documented that treatment with QE restored serum biochemical parameters towards normal levels, decreased levels of lipid peroxidation and increased levels of reduced glutathione and other antioxidant enzymes (CAT and GPx) on HepG2 cell line induced liver cancer in rats, indicating that QE exhibited antiproliferative effect by modulating hematological parameters, lipid peroxidation and augmenting antioxidant defense system in proliferation bearing rat (Rani, 2017). In addition, QE could attenuate invasion and metastasis of HCC by downregulating ras homolog family member C level and patientderived xenografts by targeting specific E3 ubiquitin protein ligase 2 (Huang et al., 2024). Recently, QE/anti-PD-1 antibody combination therapy reshaped HCC tumor microenvironment in mice in parallel with regulating the gut microbiota and macrophage immunity, suggesting that traditional Chinese medicine ingredients in combination with PD-1 inhibitors could be a potential new strategy for HCC treatment (Wu et al., 2023). However, QE-induced mitochondrial fusion and Pink1/Parkin-dependent mitophagy may negatively influence its anticancer effects in HCC and targeting mitophagy may enhance the therapeutic potential of QE in HCC treatment (Chen, 2024).

### 2.8 Other compounds of flavonoids

A few literatures have confirmed that rose flavonoids (RF) had good effect on liver protection. RF could destroy HepG2 cells by upregulating the expressions of pro-apoptotic proteins p53 and Bax, and downregulating levels of pro-caspase 3 and pro-caspase 9, as well as inhibiting the expression of antiapoptotic protein Bcl-2, therefore promoting release of caspase 3 and caspase 9 (Zhang Z. et al., 2022). Another study in 2013 indicated that oral administration of the total flavonoids (TFs) from Rosa laevigata Michx fruit decreased blood lipid and glucose levels and ameliorated hepatic fat accumulation through suppressing fatty acid synthesis, promoting fatty acid b-oxidation together with resisting oxidant stress and protecting mitochondria, indicating that TFs provides novel therapeutic potential against HFD-induced NAFLD (Zhang et al., 2013). Present report suggested that green tea and its constituents (rich in the flavonol group of polyphenols) offer the strong herbal therapy in liver anomalies. A large number of polyphenols with the effective galloyl group, several instaurations, hydroxyl groups and other structural specialty attribute to its antioxidant, antiinflammatory, anti-proliferative, anti-mutagenic/anti-carcinogenic potentials (Maiti et al., 2019). Prunella vulgaris total flavonoids have been confirmed to have an obvious antihepatocarcinoma effect, and the mechanism may be linked to the inhibition of autophagy which may be related to activation of the PI3K/Akt/mTOR pathway (Song et al., 2021).

## 3 Clinical trial of flavonoids in liver diseases

This is supported by Tehrani et al.‘s work demonstrating that soy isoflavones could significantly reduce triglyceride, low density lipoprotein, total cholesterol, waist circumference and hip circumference in NAFLD patients in a randomized clinical trial involving 50 patients with NAFLD (Neshatbini Tehrani et al., 2024). In a parallel randomised, double-blind clinical trial study (Namkhah et al., 2021), NIN capsules (100 mg) consumption significantly reduced percentages of NAFLD grades, as well as, serum levels of triglyceride, total cholesterol, and low-density lipoprotein and increased serum level of high-density lipoprotein compared with the control group in overweight/obese patients with NAFLD. Numerous clinical trials of SL for the treatment of liver disease are underway. Ahmed et al. identified a possibly unique role of SL in mitigating HCV. In this miniature clinical trial, SL adjunct therapy was observed a decrease in menace level of liver markers such as ALT, ALP, AST, and bilirubin, improved hematological indices and oxidative markers, for instance, SOD, TAS, glutathione, glutathione disulfide, and MDA, and diminished latent viral load (Ahmed et al., 2022). These findings indicated sustainable development of flavonoids-related medicine and its great potential of clinical application for the treatment of liver diseases.

## 4 Conclusion and futuristic vision

Over the last decade, many attempts have been made to explore the hepatoprotective effects of some natural products for promoting drug discovery. Among them, flavonoids are particularly outstanding in the treatment of liver disease. The beneficial biological activities of flavonoids are undoubtedly related to their structural composition and attributes. The common flavones, isoflavones, flavanols, dihydroflavones, dihydroflavonols, anthocyanidins and chalcones, as part of the daily diet, imparts health benefits such as antioxidant, anticancer, antiinflammatory, and antihepatic fibrosis properties, which is a better choice for development as a therapeutic liver.

**FIGURE 4:**
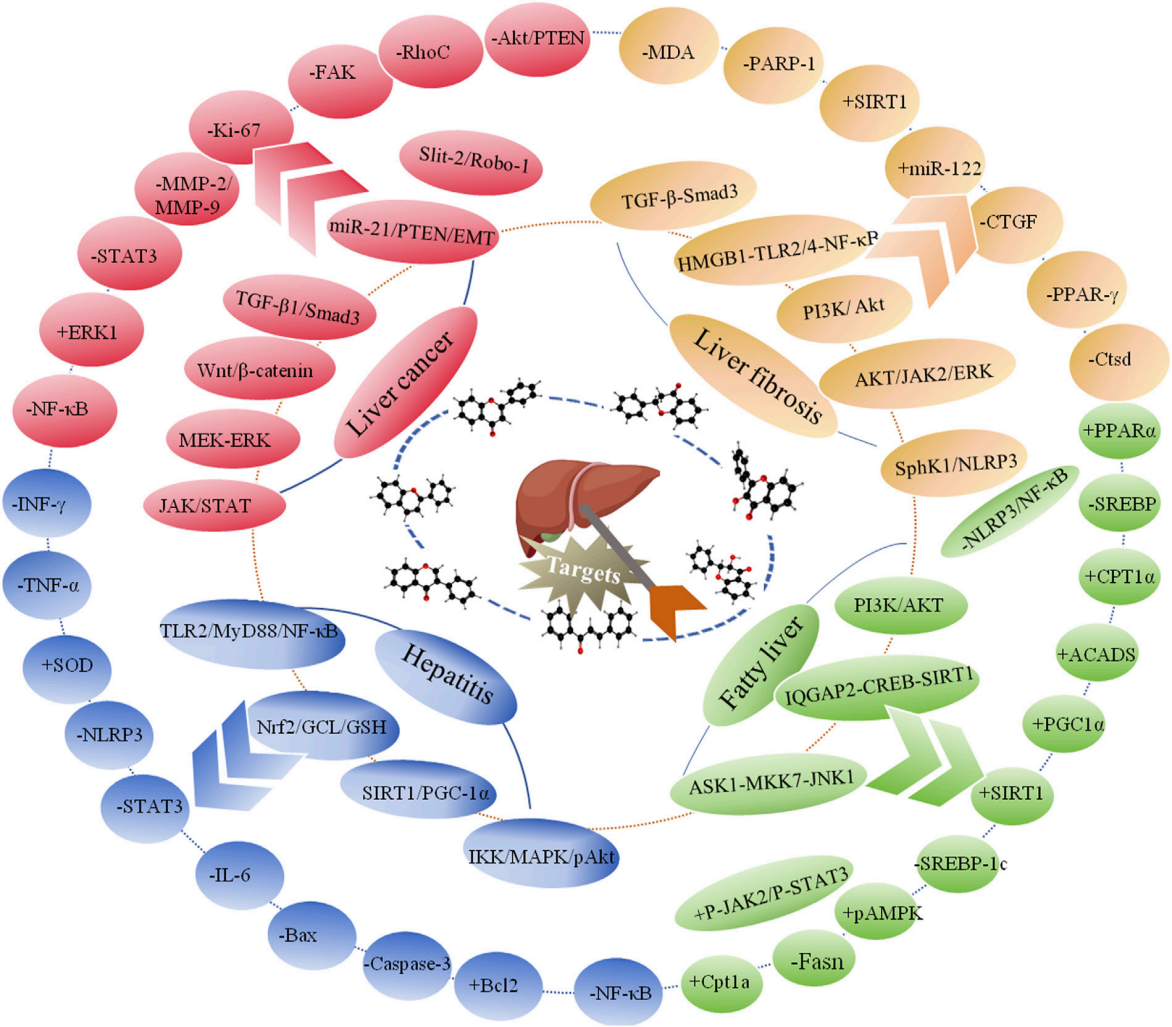
The interaction network mechanism of flavonoids against liver diseases. +: upregulation, −: downregulation.

In this review, we summarize the main network mechanisms of action of seven classes of flavonoids with potential hepatoprotective functions (Figure 4). For instance, the activation of the Nrf2/HO-1 pathway, the downstream upregulation of antioxidant enzymes, including SOD, GST and GPx, and the increased intracellular levels of glutathione have been proposed as common mechanisms underlying the antioxidant effects of these compounds. Although almost all these flavonoids could improve hepatic oxidative stress, liver inflammation and lipid metabolism regardless of their etiologies, the minimum effective dose and efficacies vary considerably among diseases. The varying therapeutic efficacies of these flavonoids is largely due to their unique chemical structure or their binding to different target receptors. The therapeutic effects of flavonoids in liver diseases are mainly based on their antioxidant capacity, which depends on the molecular configuration, the position and number of hydroxyl groups. Exactly, the3′− 4′catechol structure in ring B is a strong indicator of antioxidantability along with a hydroxyl group on the 3-position of ring C (Ravishankar et al., 2013). QE, for example, has superior antioxidant activity thanks to specific functional groups in its molecular structure. In contrast, SL has forged a path to triumph in the treatment of viral hepatitis, mainly due to its ability to block viral replication by enhancing the JAK-STAT antiviral signaling pathway. In the area of antihepatic fibrosis, ISL could inhibit Annexin A2 expression and block the sphingosine kinases/sphingosine-1-phosphate/IL-17 signaling pathway (Liu et al., 2023), yet, QE decreased hepatic mRNA levels of SphK1 and NLRP3 (Salama et al., 2024). Certainly, the study of the pharmacological mechanism of single compounds in Chinese medicines is an inherently complex endeavour and is yet to be further confirmed. Overall, the pharmacological mechanisms of flavonoids are almost the same for the basic structure is consistent, while the targets of action are slightly different due to the number and position of substituents, and hopefully, there may be new surprises waiting to be discovered.

It is a complex and long-term study due to the heterogeneity of molecular structures. Several obstacles have limited the clinical application of flavonoids and delayed the discovery of new therapies based on flavonoids. One of the most concerning issues that arise is their bioavailabilityand absorption after oral ingestion. For its development as a viable treatment option against liver diseases, preclinical and clinical studies, investigation of various drug formulations and routes of administration, development of new solubilizing formulations (such as lipid-based nanoparticles, cyclodextrin-inclusioncomplexes, nano-emulsions and others arebeing) or structural modifications of compounds will also have to be carried out. For instance, a study in 2019 indicated that plasma levels of SL were higher after the administration of SL-phosphatidylcholine complex capsules compared with that after conventional SL tablets in healthy volunteers (Méndez-Sánchez et al., 2019). Our research has also been devoted to the development of novel nanoformulations of flavonoids (Liao et al., 2020). Literature data suggested that despite encouraging preclinical data, other promising compounds, such as GEN, QE are still waiting for further clinical confirmation or more evidence of efficacy. Moreover, the combined application of flavonoids seems to be a promising method for liver diseases treatment. The combination of QE with rosuvastatin, PA with 5-fluorouracil, sorafenib and AP, and baicalin capsules with adefovir has been explored in previous studies. In summary, there remain three bottlenecks for clinical breakthroughs, as follows, the low bioavailability of natural products should be improved, the combined application of two or more compounds should be investigated for more prominent pharmacological effects and the evidence of clinical efficacy of other compounds requiring further confirmation. Besides, although flavonoids are already relatively safe compounds, the toxicity issues need to be further explored with a view to obtaining a safe and effective dose range. In summary, even after taking these obstacles into account, the flavonoids, including SL, AP, ACN, GEN, PA, ISL and HD, are valuable drug candidates for the treatment of various liver diseases that have attracted extensive attention for their significant antioxidative stress, antiinfammatory, antifibrosis antilipotoxicity, and anticancer effects. In our opinion, the advancement of pharmacology and toxicology researches of natural products and their derivatives will further contribute to the development of flavonoids, which still show great potential to become the promising therapeutic options to improve liver diseases.
